# Cost-utility analysis of case management for frail older people: effects of a randomised controlled trial

**DOI:** 10.1186/s13561-015-0051-9

**Published:** 2015-05-30

**Authors:** Magnus Sandberg, Ulf Jakobsson, Patrik Midlöv, Jimmie Kristensson

**Affiliations:** 1 Department of Health Sciences, Faculty of Medicine, Lund University, P.O. Box 157, SE-221 00, Lund, Sweden; 2 Center for Primary Health Care Research, Faculty of Medicine, Lund University, SE-205 02 Malmö, Sweden; 3 Department of Clinical Sciences in Malmö, Faculty of Medicine, Lund University, SE-205 02 Malmö, Sweden

**Keywords:** Case management, Older people, Frail, Healthcare costs, Informal care, Cost-utility analysis

## Abstract

**Background:**

To evaluate the effects of a case management intervention for frail older people (aged 65+ years) by cost and utility.

**Materials and methods:**

One hundred and fifty-three frail older people living at home were randomly assigned to either an intervention (n = 80) or a control group (n = 73). The 1-year intervention was carried out by nurses and physiotherapists working as case managers, who undertook home visits at least once a month. Differences in costs and quality-adjusted life years (QALYs) based on the health-related quality-of-life instruments EQ-5D and EQ-VAS, and also the incremental cost-effectiveness ratio were investigated. All analyses used the intention-to-treat principle.

**Results:**

There were no significant differences between the intervention group and control group for total cost, EQ-5D-based QALY or EQ-VAS-based QALY for the 1-year study. Incremental cost-effectiveness ratio was not conducted because no significant differences were found for either EQ-5D- or EQ-VAS-based QALY, or costs. However, the intervention group had significantly lower levels of informal care and help with instrumental activities of daily living both as costs (€3,927 vs. €6,550, p = 0.037) and provided hours (200 vs. 333 hours per year, p = 0.037).

**Conclusions:**

The intervention was cost neutral and does not seem to have affected health-related quality of life for the 1-year study, which may be because the follow-up period was too short. The intervention seems to have reduced hours and cost of informal care and help required with instrumental activities of daily living. This suggests that the intervention provides relief to informal caregivers.

## Background

Case management for frail older people is an intervention that has been introduced to address problems with fragmented care and discontinuity, improve older people’s functional status and reduce healthcare use [1]. Despite being a part of the health system for at least 40 years, there is no agreed definition of case management [2, 3]. It has been suggested that basic case management includes: identification and outreach, comprehensive individual-based assessment, care planning, care coordination, service provision, monitoring, evaluation and meeting individual needs [1, 4]. Effects have been demonstrated on both functional status and healthcare use [5] and case management has also been experienced to give trust, continuity and security, as well as a mutual confidence between participants and case managers [6]. When conducting healthcare interventions, economic evaluation is important to be able to determine their cost effectiveness [7]. This provides decision makers with valuable information to help them prioritise interventions and allocate money to different areas of healthcare, as well as to understand the cost consequences of healthcare interventions [7]. This makes economic evaluations of case management interventions important, and it has been suggested in a recent study that larger studies capturing patient outcomes as well as costs are required [8]. Studies have rarely evaluated costs in relation to utility outcomes that reflect the effects on the participants. Comprehensive health economic evaluations looking at both costs and utility gained for the individual are therefore necessary to determine whether an intervention should be implemented.

Case management interventions contain several components that may operate both independently and interdependently. This, together with the behaviours required by those delivering or receiving the intervention, and the number and variability of outcomes, means that case management can be defined as complex. It could also be challenging to evaluate [9]. To deal with these challenges, the British Medical Research Council has developed a framework for evaluating complex interventions [9] that suggests a multi-step approach including a development phase, feasibility/piloting, evaluation (preferably through a randomised controlled trial) and implementation. Its guidance also states that an economic evaluation is important to determine cost effectiveness of a complex intervention and whether it should be implemented.

One goal of case management is to make sure that the participants have their needs met and so get access to appropriate healthcare. It is argued that economic evaluations should preferably take the societal perspective [7], which means that everyone affected by an intervention, and all associated costs, should be counted regardless of who bears those costs. The choice of perspective may be particularly important for organisational interventions including case management as it may affect resource use both in the health sector and in other sectors, including other public sector bodies and informal care [10]. Few studies have investigated the effects of case management for older people on healthcare costs, and the results are inconclusive. Most studies are unable to show significant results, some show significantly lower costs in the case management group, but others show costs of case management that are significantly higher [11–17]. Reasons for these inconclusive results may be differences in the quality of the studies, content of the intervention, that the studies were performed in different settings/populations, and with different types of costs included in the calculations. Unless they included the same set of costs, e.g. combinations of inpatient and outpatient costs, costs for municipal care and services, and costs for informal care, the results are not completely comparable. All healthcare and other costs should therefore be included for a complete health economic evaluation.

However, healthcare costs should not be the only endpoint. Studies looking at monetary effects often do not take into account potential effects on the participants. Frail older people may find it helpful to have a case manager address a previously unmet healthcare needs [18] even if this means an increase in healthcare costs. Evaluations of case management interventions focusing on benefits to the individual as well as healthcare costs are, however, lacking. If the cost for an intervention is not significantly higher than for standard care, an intervention may still be considered effective, and therefore interesting, if it has effects on, for example, quality of life (QoL). Changes in an individual’s status in relation to costs are therefore an important aspect when conducting health economic evaluations and determining whether an intervention is successful or not. Few studies evaluating case management, or interventions with similar features, have this approach, and in those that exists healthcare costs have been related to quality of care [19], death rates [20], or successful treatment [21]. All these studies are forms of cost-effectiveness analysis (CEA) and presented a health effect as a single unvalued outcome, measured in physical units related to the objectives of the programme [7]. This makes it difficult to compare and determine which is the most effective.

Another approach is the cost-utility analysis (CUA) where the outcome is usually measured in quality-adjusted life years (QALYs) [7]. The outcome of a CUA may be single or multiple, but will be generic as opposed to program specific, and incorporate the notion of value. A QALY could be described as a quality adjustment weight for a health state multiplied by the time in the state [7]. One advantage of QALYs is that when health-related quality of life (HRQoL) is measured using, for instance, SF-12 or EQ-5D, this can be converted to QALYs. Thus, QALYs can be used to measure simultaneously both changes in quantity of life (mortality) and changes of QoL (morbidity) [7]. This gives them a broad applicability and makes it possible to compare different interventions in different areas. CUA is therefore more useful to decision makers than CEA [7]. When conducting economic evaluations of healthcare interventions such as case management, it is therefore crucial to incorporate the effects of the intervention on participants’ health, and not only monetary effects. A CUA could both determine potential benefits for the individual and provide decision makers with valuable information that could help them to prioritise interventions within the healthcare sector. However, to our knowledge, no CUA of case management for frail older people has been conducted.

The aim of this study was to evaluate the effects of a case management intervention for frail older people (aged 65+ years) by costs and utility.

## Method

The study was designed as a two-armed randomised controlled trail (RCT) [22]. Details of this have been published previously [23, 24].

### Setting

The health system in Sweden is highly decentralised and healthcare and social services are provided mainly by 20 county councils and 290 municipalities. Care and services in Sweden are based on a welfare system and are mainly funded by taxation [25]. Long-term care and social services are provided by the municipalities either at home or in special accommodation such as nursing homes. Long-term municipal care could include tasks such as help with cleaning, doing laundry, help with shopping, personal care, transport services, meals on wheels, and provision of personal safety alarms and is provided in the older person’s home or in special accommodation [26]. The municipalities can also provide healthcare and are responsible for nursing home care [26]. Home care from physicians, together with healthcare, treatment, rehabilitation and specialised medical care in inpatient, outpatient or primary care centres, is provided by the county councils.

The study municipality was medium sized with approximately 30,000 inhabitants in 2007. It contained both rural and urban areas as well as industrial and agricultural environments. The nearest hospital was approximately 20 km from the main municipality town. The hospitals are responsible for all inpatient care and, with private specialist healthcare clinics, outpatient specialist care. Public or private primary care centres are responsible for all primary care. The municipality in this study had three primary care centres and a private specialist healthcare clinic with medical services including gynaecology, general orthopaedics, day surgery and physiotherapy.

### Sample

**Fig. 1:**
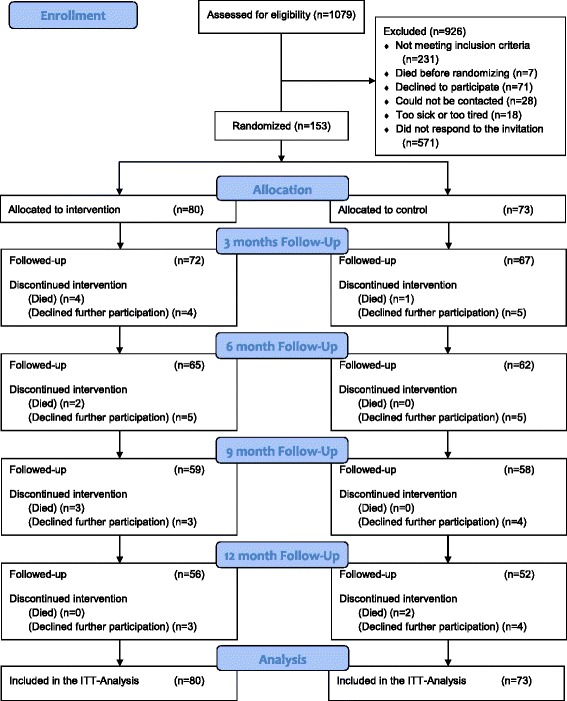
Consort 2010 flow diagram

Between October 2006 and April 2010, 153 participants were consecutively recruited. Criteria for inclusion were: (1) aged at least 65 years, (2) resident in an ordinary home, (3) self-reported help with two or more activities of daily living, and (4) had been admitted to hospital at least twice, or paid at least four visits to outpatient care, in the 12 months before entering the study. The activities of daily living were defined as the 10 activities in KATZ ADL-index [27], and an additional question about if they needed help with administration of pharmaceuticals. There was also an open question were the participants could give another activity for which they needed help and was a part of their everyday life. The person was regarded to be dependent regardless of who gave the help, i.e. municipality, private organisation, husband/wife, relatives, friends etc. Those not able to communicate verbally, whit a cognitive impairment with a score of <25 on the Mini Mental State Examination (MMSE) [28] before the start of the intervention, were excluded. Those or who moved to special accommodation during the study or had MMSE score blow 25 for two consecutive data collections time points was regarded as dropout. The participants were recruited from a university hospital (n = 20), from primary care centres in the study municipality (n = 117), through the municipal home care organisation (n = 13) or because they contacted the research group (n = 3). Potential participants were approached direct by staff at the different settings or by mail or telephone in different screening procedures in the hospital and in primary care. At the hospital wards were screened for older people living in the chosen municipality and whether or not they had an additional admission in the last year. At the primary care centres the medical records were screened for all patients with four or more visits in the last year. Participants were asked, either in person, by telephone, or by mail, whether or not they would allow someone in the research group to contact them for information about the study and to see if they met the inclusion criteria. A total of 1,079 were contacted, of whom 862 by telephone or mail in some type of screening procedure. Of those approached, 926 were excluded (231 did not meet the inclusion criteria and 688 could not be randomised) (Fig. 1). The most common reason for this was not answering the invitation letter about wanting to be contacted by the research team (n = 571). Those included were randomly allocated to the control group (n = 73) and received standard care or to the intervention group (n = 80), who also received a case management intervention. One hundred and six completed the 1-year study (Fig. 1). All participants provided informed written consent.

### Intervention

This study was developed in line with the first version of the Medical Research Council’s framework for development and evaluation of RCTs for complex interventions to improve health [29]. Details of the pilot study of the RCT, including information about the development and content of the intervention, have been published previously [23]. The intervention changed slightly after the pilot phase, in that physiotherapists were also employed as case managers. More details about the interventions as well as evaluation of healthcare use have been published elsewhere [24]. The providers’ and the receivers’ experiences of the case management intervention have also been published [30].

The case management intervention consisted of four dimensions: traditional case management tasks (assessment, care plans, care coordination, home visits, telephone calls, and advocacy), general information (about the healthcare system, social activities, nutrition, exercise), specific information (related to the respondent’s specific health status, individual needs and medication) and aspects related to safety, with the CM being contactable by cell phone during working hours. The part of the programme carried out by the case managers with a nursing background focused more on nursing care, on the participant’s health and on making and evaluating a care plan, while the case managers with a background in physiotherapy focused more on the prevention of falls (balance training, home adaption, aids) and on increasing physical function. The participant received at least one visit per month from each of two CMs, one with a nursing background and one with a physiotherapy background, during the 1-year intervention, but they could do the visits together at the same time. The visits took place in the participant’s own homes or, for instance, in hospital if they were hospitalised at the time.

For those completing the intervention, the nurse case managers made on average 11.1 home visits and 1.9 telephone calls during the 12-month intervention. The corresponding numbers for the physiotherapist case managers were 10.4 visits and 0.8 telephone calls. For those dropping out of the study (Fig. 1), the mean intervention time was 5 months and they received an average of 3.7 visits and 1.0 telephone call from the nurse case manager and 2.5 visits and 1.0 telephone call from the physiotherapist case manager.

Both groups received ordinary care and service as usual described in the Setting section, which means that the intervention was given in addition to this existing health system. Ordinary care could include some of the parts of the intervention, for instance information about medications, but it was assumed that the intervention could perform this better by doing it in a more structured and comprehensive way, with a high degree of continuity and person-centeredness. With a high degree of continuity the case manager model focus on assessment – care plan – reassessment, and care coordination.

### Data collection

Researchers, working independently of those delivering the intervention, carried out structured interviews at baseline and every third month during the 1-year intervention. The interview included questions and various tests, such as a balance test. The questions covered background data, social aspects, health status, HRQoL, life satisfaction, care and services, balance and physical activity, informal help with instrumental and personal activities of daily living (ADL), municipal care and services and home healthcare [23, 24]. Baseline characteristics in the present study included demographics, self-reported health complaints in the last 3 months [31], functional status assessed with the ADL staircase [32], risk of depression assessed with the 20-item version of the Geriatric Depression Scale [33] and cognitive status assessed with the MMSE [28].

The different costs are presented in three categories; health sector, other sectors, patient and family as suggested by Drummond et al. [7], and for the intervention. All costs are presented in 2011 prices and transformed from Swedish Kronor (SEK) to Euros (€) using the mean exchange rate for 2012.

#### Health sector resource use and prices

Healthcare costs for inpatient and outpatient care provided by the county were collected from the Patient Administrative Support in Skåne (PASiS). PASiS is a register for all publicly-organised inpatient and outpatient healthcare in the region. It includes individual data for all healthcare use and is used by the county for calculations of healthcare costs. The Swedish southern regional healthcare organisation has listed six different principles that are used, alone or in combination, for deriving healthcare costs [34]. These are: (1) price per diagnosis related group (DRG) per visit or care event, (2) price per product, (3) price per patient for special or extreme care events, (4) price per admission/care event/physician visits/medical treatment (5) price per bed day, and (6) price per subscription or a fixed price for all patients attached to a health centre. The main principle has been DRG and particularly the Nordic version (NordDRG) [35]. DRGs are a system classifying cases into categories with similar resource use. Grouping is made based on diagnosis, procedures performed, age, sex and status at discharge [36]. Each DRG has a specific price that is used for reimbursement. Costs for private care were collected from PrivatStat, which is a register for all private outpatient healthcare. The costs for each patient contact are calculated in similar ways as for public care. However, each private healthcare clinic is reimbursed for how many, and the characteristics of, registered patients at the clinic. The reimbursement also depends on each clinic’s contract with the county, and is therefore not possible to calculate. The costs for private healthcare are therefore somewhat underestimated.

#### Other sectors’ resource use and prices

Community care costs, or costs provided by the municipality, were calculated with self-reported estimates. Numbers of hours of municipal home care services were collected for daytime help with Instrumental Activities of Daily Living (IADL), Personal Activities of Daily Living (PADL), evening/night municipal home care services (both IADL and PADL) and number of hours each week of accompanied travel services, or being accompanied on visits, for example, to doctors’ appointments. The costs for municipal home care services have been reported to be approximately €30 per hour for IADL in the studied municipality, including accompanied travel services, and about €42 for PADL, and these figures were used in this study. If the person lived in a rural area, an additional cost of €4 was added. According to the municipality, these figures included all costs for the provision of the services. Self-estimates of numbers of grocery deliveries per week, whether they had a personal safety alarm, whether they had spent any time in short-term accommodation since last contact, and if so, how many days, were also collected. In this municipality, people with grocery deliveries were assigned 30 min extra home care service if they lived in an urban area and 60 if they lived in a rural area. The same hourly cost as for IADL, €30, was used by the municipality for accompanied travel services. Again, an additional €4 was added for people living in rural areas. The municipality provided details of the number of registered safety alarms as at 31 December 2011, together with the total costs for safety alarm services in 2011, which formed the basis of a monthly cost per safety alarm of about €36. The municipality also provided information on total costs and use of short-term accommodation for 2011, resulting in an estimated daily cost of €207.

Data concerning home healthcare provided by the municipality was also linked to self-reported estimates from the structural interviews. The participants were asked to estimate the number of hours of home healthcare they received each week, the number of visits each month, and how many visits from each of the professions, physician, nurse, nurse assistant, physiotherapist or “other”. There was, however, no question about the amount of time per profession, and time was assumed to be evenly distributed between the professions. Participants who estimated that they had received visits totalling less than 1 hour per week were rounded up to one. Part-values above one were rounded to the nearest integer. Costs were calculated using the average hourly salary, including payroll surcharge, in the municipality. The hourly costs for nurse assistant/other, registered nurse, physician, and physiotherapist were estimated as €25, €33, €64 and €30 respectively [37].

#### Participant and family resource use and prices

Costs associated with the participant and family included the patient fee and estimated costs for informal help provided by family and friends. Patient fees were registered for each participant in PASiS and PrivaStat and were collected from these registers. Data concerning informal care was estimated using the opportunity cost method [38]. Number of hours of informal care with IADL and PADL were collected through the structural interviews. For both IADL and PADL, the participants were asked to estimate how many hours of help they received each week and from whom. Responses from participants with values below one but above zero were rounded up to one. Part-values above one were rounded to the nearest integer. The reported number of hours for each month was multiplied by the average hourly salary in the studied municipality (~€20, including payroll surcharge) [39].

#### Intervention costs resource use and prices

The costs for the intervention were calculated using the case managers’ salaries. In general, each case manager was employed to work 50 % full time, but this could vary during the study period depending on the number of employed case managers and the case load. A 50 % whole time equivalent was used to estimate the costs of each case manager’s salary. The case managers also supported a cross-over group and the proportion of participants in this study, and thus the cost for each month was calculated. The costs for each month during the study period, from September 2006 to April 2011, were summed, and this was used as the basis for a mean cost per participant in the intervention group. The cost for each participant for a 3-month intervention period was estimated as €879.

#### Effect measurement

The outcome measurement used in this economic evaluation was QALY derived from the instrument EQ-5D measuring HRQoL [40]. EQ-5D is a non-disease specific instrument with five three-level questions covering five dimensions: mobility, self-care, usual activities, pain/discomfort and anxiety/depression [41]. This means that there are 243 possible health states (3^5^) and each health state can be valued through different methods to obtain a numeric value, a QALY, on a 0–1.00 scale, with 1.00 indicating “full health” and zero representing “dead” [7]. For this study, the values or weights used for all health states were taken from a study with a British general community sample (n = 3,395) using a time-trade-off technique for direct valuation on 42 of them [42]. Some health states can obtain values below zero, meaning that the health state is considered worse than death [42]. The instrument has been well used and has proven validity and reliability. Together with the five-dimensional scale, there is also a visual analogue scale, EQ-VAS [41]. On this scale, the respondents are asked to mark off their own perceived health state on a 20-cm vertical 100-step scale, where the endpoints are zero and 100 and labelled “worst imaginable health state” (zero) and “best imaginable health state” (100) [41]. EQ-VAS scores were normalized (i.e. divided by 100) to obtain scores from 0 to 1.

### Statistical analysis

The control group and case management group were compared for background data at baseline using the chi-square test for nominal data, Mann–Whitney *U*-test for ordinal data and the Student’s *t*-test for interval/ratio data. As the structured interviews were every third month, the values for the participant and family-related costs, and costs in other sectors were those for the preceding 3 months. Costs for the 1-year study period were calculated using the 3-, 6-, 9- and 12-month follow-up. One-year costs were also calculated for health sector costs (publicly and privately organised inpatient and outpatient care). Health sector costs for the year before entering the study were used to calculate a 3-month average, which was used as baseline cost. The data were analysed on the intention-to-treat principle (ITT) [43]. For missing values in healthcare costs from the region (inpatient and outpatient care costs), those dropping out were attributed their mean healthcare cost for the time from the year preceding their inclusion in the study to the time they dropped out, i.e. case mean substitution [44]. Costs for home safety alarm imputations were made using their last known value, i.e. last observation carried forward [45]. If no known value existed, it was assumed that the individual did not have an alarm. For individuals with one or more missing values between baseline and final value, linear interpolation was used for imputation. All other imputations were made with case mean substitution [44] using the individual’s known values for the other observations. If no valid values existed, the group mean was used. A complete case analysis was also conducted [46]. Differences between those completing the study and attritions, i.e. the loss of participants over the course of the study and which can create bias by changing the composition of the sample initially drawn [47], was analysed. A total cost for the 1-year intervention meant that the baseline costs were not included. QALYs were calculated for the 1-year period using the area under the curve technique, giving a mean value using all five measurement points [43]. To illustrate the effect for each individual, the difference between baseline and 12-month values for both QALYs and total costs were calculated for each individual and plotted in a scatter plot. The incremental cost-effectiveness ratio (ICER) was calculated as: $ICER=\frac{\left({C}_{CM}-{C}_C\right)}{\left(QAL{Y}_{CM}-QAL{Y}_C\right)}=\frac{\Delta C}{\Delta QALY}$

To reduce the risk of over- or underestimations of costs per hour for informal care, municipal care, and home healthcare sensitivity analyses were conducted with the costs set to half, and double the amount used in this study, respectively.

## Results

No significant differences between the case management group and the control group were seen in demographic characteristics at baseline (Table 1). There were no significant differences between the case management and control groups in the number of self-reported diagnosis groups or self-reported health complaints, functional dependency, the risk of depression or cognitive impairment at baseline (Table 2). There were also no significant differences in the use of municipal care and services, home healthcare or informal care during the 3 months prior to baseline (Table 2) or costs in the health sector (inpatient care: €1,773, SD €4,052 vs. €1,659, SD €2,334, p = 0.834; outpatient care: €714, SD €750 vs. €718, SD €638, p = 0.971 in intervention and control group, respectively).

**Table 1:** Demographic information and socioeconomic status at baseline

Group	CM (n = 80)	Control (n = 73)	p-value
**Demographics**			
Age, mean (SD)	81.4 (5.9)	81.6 (6.8)	0.795^a^
Women, n (%)	52 (65.0)	50 (68.5)	0.648^b^
Municipal care at baseline, n (%)	30 (37.5)	24 (32.9)	0.550^b^
Marital status, n (%)			0.338^b^
Married or living together	23 (28.8)	29 (39.7)	
Widow/er	41 (51.3)	34 (46.6)	
Divorced or living apart	8 (10.0)	7 (9.6)	
Other	8 (10.0)	3 (4.1)	
Having children, n (%)	67^†^ (84.8)	67 (91.8)	0.184^b^
**Socioeconomics**			
Educational level, n (%)			0.437^c^
Primary <8 years	40 (50.0)	31 (42.5)	
Secondary >8 years	32 (40.0)	35 (47.9)	
Third level/university	8 (10.0)	7 (9.6)	
Financial status, n (%)^‡^			0.477^c^
Better than others	16 (21.1)	10 (14.7)	
Same as others	51 (67.1)	50 (73.5	
Worse than others	9 (11.8)	8 (11.8)	

^†)^Missing = 1

^‡)^Missing: Intervention group = 4, Control group = 5

^a)^Student’s *t*-test

^b)^Chi-squared test

^c)^Mann–Whitney *U*-test

*CM* Case management

**Table 2:** Self-reported diagnosis groups, self-reported health complaints, ADL, risk of depression, cognitive impairment, and use of care and services at baseline

Group	CM (n = 80)	Control (n = 73)	p-value
**Self-reported diagnosis groups**			
Number of diagnosis groups, median (q1-q3)	3 (2–4)	4 (3–5)	0.163^a^
Range	1-8	1-7	
**Self-reported health complaints**			
Number of complaints, median (q1-q3)	11 (7–15)	11 (8–15)	0.655^a^
Range	2-22	2-23	
Five most common complaints, n (%)			
Walking problems	55 (68.8)	55 (75.3)	0.365^b^
Pain in the musculoskeletal system	55 (68.8)	52 (71.2)	0.738^b^
Breathlessness	47 (58.8)	40 (54.8)	0.622^b^
Fatigue	45 (56.3)	41 (56.2)	0.991^b^
Memory impairment	41 (51.3)	42 (57.5)	0.436^b^
**Activities of daily living**			
Dependency in no. of ADL activities, median (q1-q3)			
IADL	2 (1–3)	2 (1–3)	0.651^a^
PADL	0 (0–0.8)	0 (0–0.5)	0.881^a^
Total ADL	2 (1–3)	2 (1–3)	0.831^a^
**Risk of depression**			
GDS-20, median (q1-q3)	6.0^†^ (3.0-8.0)	6.0 (4.0-8.0)	0.824^a^
**Cognitive impairment**			
MMSE, median (q1-q3)	28.0 (27.0-29.0)	28.0^‡^ (27.0-29.0)	0.571^a^
**Use of care and services during the three months prior to baseline**			
Municipal care and services, mean (SD)			
Hours of help with IADL^§^	4.2 (10.8)	2.9 (6.3)	0.372^c^
Hours of help with PADL^§^	3.5 (11.3)	5.9 (25.2)	0.483^c^
Hours of help at night^§^	1.7 (8.9)	0.0	0.118^c^
Number of deliveries of groceries^§^	1.5 (6.9)	4.0 (16.1)	0.235^c^
Hours of accompanied travel services^§^	0.2 (1.6)	0.2 (1.6)	0.975^c^
Days at short time stay^§^	0.2 (1.7)	0.0	0.321^c^
Proportion with personal safety alarm, n (%)	44 (55.0)	36^†^ (50.7)	0.598^b^
Home healthcare, mean (SD)			
Hours of municipal home care: daytime^§^	1.2 (4.9)	1.0 (4.1)	0.734^c^
Hours of municipal home care: evening^§^	0.0	0.4 (2.2)	0.159^c^
Hours of municipal home care: at night^§^	0.0	0.0	NA
Informal care, mean (SD)			
Hours of help with IADL	55.4 (76.1)	72.1 (103.1)	0.263^c^
Hours of help with PADL	5.4 (31.6)	2.7 (12.5)	0.496^c^

^†)^Missing = 2

^‡)^Missing = 1

^§)^Missing: intervention group = 0-11, Control group = 2-7

^a)^Mann–Whitney *U*-test

^b)^Chi-squared test

^c)^Student’s *t*-test

*CM* Case management, *NA* Not applicable

There were no significant differences between groups in the total cost for the study year, nor in costs in the health sector, in other sectors, nor associated with participant and family (Table 3). A significantly lower amount and costs of informal care with IADL was seen in the intervention group than the control group (200 hours vs. 333 hours, p = 0.037; €3,927 vs. €6,550, p=0.037) (Table 3). QALY for the entire study year did not differ significantly regardless of whether they were based on EQ-5D or EQ-VAS (Table 3). ICER calculations were not conducted because no differences were found for either EQ-5D- or EQ-VAS-based QALY, or costs.

**Table 3:** Costs, use of care and QALYs, during the 12-month study for the intervention, control groups

Group	CM (n = 80)	Control (n = 73)	p-value^a^
**Health sector**			
Costs (in €) of in and outpatient care			
Inpatient care costs in €, mean (SD)	9,319 (26,408)	4196 (6,738)	0.097
Outpatient care costs in €, mean (SD)	2,560 (2,388)	2656 (6,738)	0.800
Total costs in €, mean (SD)	11,880 (27,832)	6853 (7,585)	0.124
**Other sectors**			
Use of municipal home services, mean (SD)			
Hours of help with IADL	20.9 (48.7)	14.9 (29.2)	0.363
Hours of help with PADL	13.7 (36.3)	15.0 (52.7)	0.866
Hours of help at night	2.7 (14.6)	1.9 (7.9)	0.660
Costs (in €) of municipal home services, mean (SD)			
Help with IADL	633 (1,450)	462 (915)	0.389
Help with PADL	593 (1,551)	666 (2,414)	0.822
Help at night	114 (615)	82 (346)	0.693
Total for municipal care	1645 (3,473)	1671 (3,310)	0.961
Use of municipal home care, mean (SD)			
Hours of municipal home care: daytime	15.3 (55.8)	10.4 (34.4)	0.512
Hours of municipal home care: evening	1.1 (6.8)	3.1 (11.0)	0.194
Hours of municipal home care: at night	0.3 (2.9)	0.0 (0.0)	0.339
Costs (in €) of municipal home care, mean (SD)			
Municipal home care: daytime	355 (1,318)	241 (796)	0.523
Municipal home care: evening	24 (143)	67 (237)	0.182
Municipal home care: night	7 (61)	0 (1)	0.340
Total for municipal home care	385 (1,375)	307 (906)	0.683
Intervention costs (in €), mean (SD)	3516 (0)	NA	NA
**Participant and family**			
Use of informal care, mean (SD)			
Hours of help with IADL	**200 (324)**	**333 (445)**	**0.037**
Hours of help with PADL	23 (128)	64 (390)	0.374
Costs (in €) of informal care, mean (SD)			
Help with IADL	**3,927 (6,361)**	**6,550 (8,754)**	**0.037**
Help with PADL	457 (2,506)	1,265 (7,676)	0.374
Total for informal care	4,383 (8,311)	7,815 (14,486)	0.079
Patient fees, mean (SD)	111 (62)	115 (57)	0.651
**Total costs (in €), mean (SD)**	21,920 (32,936)	16,762 (17,064)	0.235
**QALY**			
EQ-5D based, mean (SD)	0.61 (0.25)	0.60 (0.23)	0.801
EQ-VAS based, mean (SD)	0.61 (0.17)	0.63 (0.12)	0.401

^a)^Student’s *t*-test, *CM* Case management, *NA* Not applicable

Plotted differences between baseline and 12-month costs and QALY, both EQ-5D- and EQ-VAS-based, showed no clear patterns of effects. There were no significant differences between baseline and 12-month measurements on total costs or on either type of QALY.

For EQ-5D and EQ-VAS, the response rate varied between 99 % and 66 % for different data collection time points, with the lowest response rate for the last measurement (12 months interview). For municipal care, the corresponding numbers were 99–65 %, and for informal care 100–69 %, respectively, once again with lowest on 12-months interviews. The complete case analysis showed informal care costs being significantly lower in the case management group than the control group at 3, 6 and 12 months after baseline (761 vs. 1,453, p = 0.024; 790 vs. 1,583, p = 0.045; and 705 vs. 2,113, p = 0.022 respectively), as well as for informal care costs for the entire 1-year study (2,712 vs. 5,774, p = 0.021). Apart from this, there were no other major differences. The number of attritions did not differ in the two groups (p = 0.673) and those dropping out did not differ significantly from non-attritions for age (p = 0.466), municipal care (p = 0.161), marital status (p = 0.562), having children (p = 0.386), educational level (p = 0.562), or economic status (p = 0.346) at baseline. There were no significant differences in the number of self-reported diagnosis groups (p = 0.944), the number of self-reported health complaints (p = 0.293), functional dependency (p = 0.095), risk of depression (0.495) or cognitive impairment (p = 0.215) at baseline. There were significantly more men among attritions than in the total sample at baseline (47.4 % vs. 28.7 %, p = 0.034).

## Discussion

One of the key findings in this study was that there were no significant differences in total cost for the 1-year intervention. One reason for this may be low power and related to a heterogeneous sample. According to Briggs [48], this is a common problem in economic evaluations where cost data are typically heavily skewed and therefore there are wide confidence intervals, meaning that very large differences or study samples are needed to achieve significant results. This wide range of costs has also been seen in other case management studies, for instance by Gage et al. [8]. Our study included people with very low healthcare costs, and where prevention of costs is therefore unlikely. This is in line with a study by Peikes et al. [49], which investigated 15 intervention programs in America, including improvements in care, patient adherence, communication, and targeting patients with chronic illnesses. They concluded that programs may need to target patients who are either too healthy and therefore at a low risk of hospitalisation and high healthcare costs, or too ill, where the prevention of costs is not possible. The problem with participants who are too unhealthy was also highlighted in another study [50] were they investigated the effects of guided care teams, including case management, and also failed to prove a significant reduction in costs. One of their explanations was that chronic diseases are incurable and in some cases exacerbate other problems. The study by Peikes et al. [49] reported that in one of the programs, the treatment-control differences were concentrated in cases with highest severity, approximately 30 % of the sample. In this subsample, the expenditure on the treatment group was about 20 % lower than the control group. Another reason for the absence of significant reductions in total costs may be the ability of the case managers to meet the individuals’ unmet needs. Coordination of care could also have led to a shift of costs where new caregiver contacts may have contributed to a lack of reduction in total costs, even if there were some reductions in healthcare utilisation [24]. Other studies have also shown that case management can meet previously unmet needs [18] and that this could lead to increased healthcare utilisation [51]. High-quality case management does not seem to prevent costs caused by progressive and worsening diseases, which may be the case in this study, as participants reported having diseases from as many as eight different diagnosis groups (Table 2). This may also be a result of a heterogeneous sample and low power, and therefore these results should be interpreted with caution.

Even though no significant results were found for total costs, another important finding was that case management seems to reduce informal care requirements, in terms of both hours of help and costs. There is a risk that this is a result of higher number of hours, however non-significant, in the control group also at baseline (55 vs. 72 hours for the three months preceding baseline) (Table 2). But during the study year there was a slightly decrease in the intervention group to 50 hours per 3 months, and an increase in the control group to 83 hours per 3 months, which make these results being a consequence of differences in baseline data unlikely. These results are important because of the effect itself and because the costs of informal care are often ignored in economic evaluations [52]. It may be difficult to capture the costs of family-provided or other informal care, but these costs should not be ignored, as informal caregivers often are the main providers of care to chronically ill people [53, 54]. In Sweden, it has been reported that informal care to older people has increased from 60 % of all care provided in 1994 to 70 % in 2000 [55], and it has also been estimated that hours of informal care are two to three times greater than hours of municipal care [54]. This is in line with the result in this study, where the control group had 3.1 times more informal care hours provided than municipal care, while the case management group had only 1.5. The result in this study therefore indicates that case management in some way reduces the burden and gives relief to informal caregivers. In this study, the opportunity cost method was chosen, although other studies propose alternatives such as contingent valuation and conjoint measurement for valuing informal care [10]. The opportunity cost method is, however, most often used, which may be because of its relatively straightforward application. This method has also been suggested when focus is on the care recipient rather than on informal caregivers [38]. The impact of case management on informal caregivers does not seem to have been evaluated in frail older populations. The reduction of burden has, however, been reported in a geriatric evaluation and management intervention for frail older people that contained many of the characteristics of case management [56]. In other areas, especially in older people with dementia, case management has been proven to reduce the burden on informal caregivers [57, 58]. The impact of the case management intervention on informal caregivers should therefore be given more attention and be evaluated more carefully, for example in aspects of their experiences of case management, or their QoL.

Both groups were relatively stable in HRQoL over time, which was not expected for a group of such frail older people. The reasons for this may be the presence of incurable chronic diseases, heterogeneity within the groups with both including healthier people as well as those with severe illness, and that the power calculations in this study were not based on HRQoL. There are also studies that have questioned whether EQ-5D and EQ-VAS are suitable to use in calculations of QALY, and if they are sensitive enough, especially among frail older people. Brazier et al. [59] concluded that EQ-5D should only be used with an older population if health changes were expected to be substantial, as it only contains three response alternatives for each item. Otherwise, the SF-36 [60] may be a more sensitive instrument. For EQ-VAS, there was a trend among participants to judge their health in even tens, especially around 50, as this is midway between the best and the worst imaginable health state. This could also have contributed to not being able to detect changes in HRQoL. There are studies that have showed that QALY derived from another HRQoL instrument, SF-6D [61], was two- to threefold higher than from EQ-5D, meaning that the choice of preference-based utility instrument may have a significant impact on study outcomes and conclusions [62].

There are also concerns when it comes to EQ-VAS. A study by Whynes [63] concluded that EQ-VAS was predictable by the EQ-5D health state, but also that there were other variables contributing systematically and independently to the EQ-VAS score, for instance psychological disposition, socio-demographic factors such as age and education, and clinically relevant distress. However, both EQ-5D and EQ-VAS are well-tested measurements, and there are studies showing that they have high validity and reliability, and are appropriate to use in an older population [64]. The reason for non-significant differences between groups in HRQoL may be that the case managers and the participant have to get to know each other, and make and launch a care plan, and work according to this plan, before any effects could be obtained. The 12-month study period may therefore be too short to fully capture the effects of the intervention. Even in the absence of clear and significant improvements in the intervention group, positive experiences of the intervention in this study have been reported [30]. It is therefore possible that the intervention could have had a positive impact on participants, but that these changes could not be detected within the timeframe of 12 months and with the chosen measurement.

### Strengths and limitations

One limitation and threat to the internal validity in this study could be the imputations made for attritions, which could lead to various forms of bias. One assumption for mean substitution is that missing values are missing completely at random (MCAR) [65]. Only one significant difference between attritions and the sample completing the study was found. When patient characteristics are similar between participants with and without missing data, the underlying reasons for missing values are independent of participant characteristics, and the missing values can be regarded as MCAR [47]. MCAR data are less likely to introduce bias to imputed data, whatever imputation technique is used [65]. This, together with a few differences between the ITT analysis and the complete case analysis, suggests the ITT analysis is unbiased.

The RCT design was a strength in this study. By randomising the sample, many threats to internal validity were eliminated. No differences between the intervention and control groups were found at baseline (Tables 1 and 2), suggesting that equivalent groups were established. Costs of inpatient and outpatient care were collected from the PASiS and PrivaStat registers. The validity of the Swedish inpatient registers (IPR), of which these two are part, has been investigated in a review [66]. Predictive values of 85–95 % were found for diagnoses in the IPR and medical records [66]. There is a risk that not all contacts were registered, but as the registrations form the basis for reimbursement to the different health agencies, this risk is considered small. All other costs were derived through self-estimates, which could be a threat to internal validity. Even for estimates based on information from the municipality and the literature, there are risks of over- and/or underestimations. Data of out-of-pocket expenses for municipal care and home healthcare was not available, and thus, not included in the analyses. This might have caused an underestimation of the participant and family prices and is a limitation of the current study. This underestimation is limited by the Swedish maximum out-of-pocket expenses for municipal care and home healthcare, which is the maximum amount an individual has to pay each month. This is 0.48 times the price base amount a year [67], and was about €197 each month in 2011. However, the randomisation procedure and the establishment of equivalent groups made effects of any self-reported or estimated errors equally likely to occur in the two groups and these are therefore not a threat to internal validity. In addition, the sensitivity analyses for informal care, municipal care, and home healthcare did not show any significant differences.

## Conclusion

Case management for frail older people neither increased nor decreased total costs during a 1-year intervention. There were also no effects on HRQoL over the 1-year study. The reason for this may be that the case managers and participants need time to get to know each other and to launch care plans before the intervention could start to be effective. The 12-month follow-up period may therefore be too short to capture the full effect of the intervention. However, the intervention seems to have an effect on costs of informal care, which is important because a substantial part of the care for older people is provided by informal carers. This intervention may therefore provide some relief for informal carers. Despite the absence of significant results in the CUA, therefore, the result of the cost analysis and the effects on informal care costs contribute to a better understanding of case management interventions for frail older people.
